# Account of a Male Child Born without a Brain, with the Appearances on Dissection, and Remarks

**Published:** 1815-08

**Authors:** 


*For the Medical and Physical Journal.*

*Account of a Male Child born without a Brain, with the Appearances on Dissection, and Remarks;* by N. L——.

AS Lusus Naturæ, similar to one which lately occurred in my neighbourhood, are not frequent, perhaps it may be thought deserving a place in your Journal. Of this I cannot be supposed the best judge.

A male brainless monster was born on Monday, June 26, at 12 A.M. The female considered herself eight months pregnant, with which the appearances of the fœtal being accorded. I first saw it five hours after birth, when it respired in an irregular jirking manner, about six or seven times in a minute, which they said had been equally imperfect from the time of its birth. Its extreme parts were livid, caused by the smothering to which it had been exposed for several minutes before my arrival. I instantly placed it in a more favourable situation for breathing; and, on examining it twelve hours after birth, I found the extremities of a natural colour; the breathing as before, and its pulse at the

 axilla axilla and groins eighty in the minute. It drew up its limbs when pinched, with a natural squall, the physiognomy at the same time indicating pain. Twenty-four hours after birth I saw it a third time, still in the same state. It made no attempts to suck substances placed in its mouth. To ascertain whether it had the power of deglutition, a tea-spoonful of milk was poured into its mouth, as it lay on its back; this caused instantly a suspension of respiration; its trunk became stiff from muscular contraction, and soon lividity again appeared on its extreme parts. Finding that it could not swallow, I was desirous that the respiration should be restored; and, to this end, leaned it forward, to allow the milk to run out of its mouth, making pressure at intervals on its abdomen and ribs. By these means it respired a little, which gradually bettered to two or three times in the minute, and it again squalled rather loudly; but never after regained its former perfection of the pulmonary functions. The temperature of the skin and mouth were always much below natural, being always cold to the touch. Its irides were unaffected by light. I saw it again at 12 p. m. of Tuesday, about five hours after its death; the skin, which, in the latter period of its life, had been livid, was now pale, and the limbs were flaccid. The irides were more dilated than during life.

*Anatomical structure.*—The natural skin terminated at an horizontal line drawn just above the eyes and ears round the head. The parts above were covered with a vascular mucous-like membrane: on cutting through it into the tumour underneath, I found it composed of numerous cysts, filled with a clear fluid; in the removal of these cysts much dark blood flowed; and there was discovered at the back part of the basis cranii, that portion of nervous matter to which the 6, 7, 8, 9, and 10th pairs of nerves are connected; these nerves, together with the nervus accessorius and vertebral arteries, were natural. There was no bone above an horizontal line, drawn at one-eighth of an inch above the orbitar processes of the os frontis. A ridge of bone extended from the sella turcica, in the direction of the crista galli, to the os frontis, as could be felt during life. On each side of this ridge the 1, 2, 3, 4, and 5th pairs of nerves passed through their foramina naturally. The heart, lungs, and large vessels, with the thymus, were perfectly formed. The gall-bladder was very much distended with a dark viscid ropy fluid. The urinary bladder contained only a few drops of fluid. The stomach and intestines were distended with air; in the stomach there was also some frothy mucus, and in the intestines a dark viscid matter, appearing like the fluid of the gall-bladder inspissated. The capsulæ renales were wanting.

Occurrences, like the present, are important in physiology; perhaps, even more favourable to its illustration than any experiments that have been or can be made, in order to fill up the many deficiencies left by nature, which happily so seldom affords malformations.

In physiology, although many points are plain to the mind, from an examination of the animal structure in its most common state, still many of its functions would remain unknown if comparison could not be made between the functions and structures of a variable animated nature.

In the present case, with such a deficiency of parts, sensation and volition were perfect, so accordant with the experiments of Docteur Le Gallois. To such as would say, the apparent volition was merely a general muscular contraction, in which the power of the flexors predominated, it will be sufficient to state, that the pained limb only was withdrawn from the injuring causes, while the other limbs remained quiescent. Connected also with the sensation of pain, was the natural sympathetic contraction of certain of the facial, laryngeal, and pectoral muscles. As to the respiration, it may be doubtful whether the imperfect state of its nervous system, or mechanical impediments, were the cause of its imperfection: if, however, any conclusion is allowable, it should be that the latter was the efficient cause. As the second impediment caused a lowering of the animal heat, and a diminution of two-thirds in the number of its respirations; from which it is likely the first impediment had a similar effect; if such were the case, it hence follows, that a natural temperature, with a breathing of eighteen or twenty times in a minute, might have existed at birth, and perhaps a life of five days, instead of thirty hours, have been its fate, as in the case of monstrosity examined by Mr. Lawrence.

As I do not feel inclined to intrude too much on your valuable pages, I will limit my pen. Allow me, however, to ask a few short questions, which are also connected with the nervous system:—In hemiplegia, is the tongue generally paralysed on the same or opposite side with the trunk, face, and pharynx? My observation, it is proper to state, favours the latter; which, if most general, your correspondents will oblige me by stating how it can be accounted for?

*Cornwall;* N. L———.

*July* 6*th*, 1815.

